# Seismic Risk Analysis Based on Strain Energy Accumulation in Focal Region

**DOI:** 10.6028/jres.099.040

**Published:** 1994

**Authors:** Motoyuki Suzuki, Yoshio Ozaka

**Affiliations:** Civil Engineering Department, Tohoku University, Aoba Aramaki, Sendai 980, Japan

**Keywords:** earthquake, extreme value distribution, magnitude, maximum ground acceleration, nonstationarity, strain energy accumulation in focal region

## Abstract

The object of this paper is to propose a stochastic method for evaluating the magnitude of future earthquakes taking account of nonstationarity in earthquake occurrence. For this purpose, the strain energy accumulation in the focal region was estimated by means of the earthquake data of the past 100 years in Japan. Furthermore, the distributions of maximum ground acceleration were derived by means of the attenuation law. As a result, we found that the distributions of maximum ground acceleration fit the type III extreme value distributions and that the expected values of those distributions depend on the strain energy accumulation significantly. Finally, it is pointed out that the nonstationarity in earthquake occurrence should be taken into consideration in order to evaluate the earthquake load in design.

## 1. Introduction

Since Japan is located on the subduction zone of a few plates, seismicity is active and many structures have been damaged during large earthquakes. To evaluate the characteristics of earthquake load in design it is important to develop a highly accurate method for estimating the ground motion within the service life of a structure.

Both deterministic and probabilistic methods are available. The former methods estimate the ground motion by means of the dislocation model. Suzuki and Satou [1] have applied this model to a great earthquake expected in the Tokai region. The latter methods evaluate the probability distribution or the expected value of recurrence of the ground motion by considering earthquake occurrence as a probabilistic event. Kawasumi [2] has proposed one such method employing cumulative frequencies. At present, it can be pointed out that probabilistic methods are more suitable for estimating the ground motion than deterministic methods, because a geophysical model and its parameters in earthquake occurrence are not known with certainty [3].

However, in traditional probabilistic methods, it is assumed that the process of earthquake occurrence is temporally stationary, i.e., that the probability of occurrence is invariant in time. Actually, it is rare for another large earthquake to occur in the same region immediately after a large earthquake. Moreover, since the service life of a structure ranges from several decades to about 100 years, it is not reasonable to assume stationarity in earthquake occurrence within the service life of a structure.

The object of this paper is to estimate strain energy accumulation in focal regions at present and to propose a new method for evaluating the ground motion.

## 2. State of the Art

Some seismic risk analyses in which the non-stationarity in earthquake occurrence is taken into consideration have been suggested. Typical models of seismic risk analyses—the time-predictable model, the slip-predictable model, and the semi-Markov model are reviewed as follows.

### 2.1 Time-Predictable Model

**Fig. 1:**
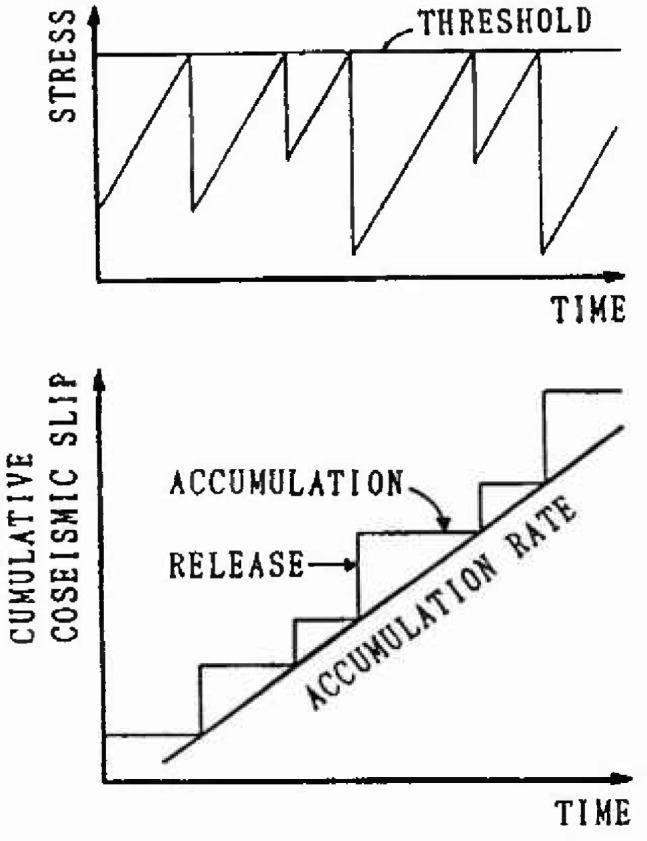
Time-predictable model.

This model was proposed by Shimazaki et al. [4]. Time history of the stress accumulation and release in a fault is represented schematically in Fig. 1. This is, stress accumulates at a constant rate up to a certain threshold, at which time an earthquake occurs and accumulated stress is released. The size of the earthquake is determined by the level of the released stress. The time when the next earthquake will occur is predictable given the size of the preceding earthquake, but it is difficult to estimate the size of next earthquake. Anagnos et al. [5] described this model by following Markov's renewal process.

$$\begin{array}{c} P\left[Y_{n+1}=J,T_{n+1}-T_{n}\leq t|Y_{0},\ldots ,Y_{n};T_{0},\ldots ,T_{n}\right]\\ =P\left[Y_{n+1}=J,T_{n+1}-T_{n}\leq t|Y_{n}=1\right] \end{array}$$ (1)

where

*I,J* = the state depending on the size of the earthquake
*Y_π_* = the state of the fault after the *n* th event
*T_π_* = the time of the *n* th event.

This means that the joint probability from present state to the next state depends only on the present state and is independent of past history. A change of stress release by measuring a coseismic slip in a fault has been proposed, because it is difficult to directly measure the level of stress release.

### 2.2 Slip-Predictable Model

**Fig. 2:**
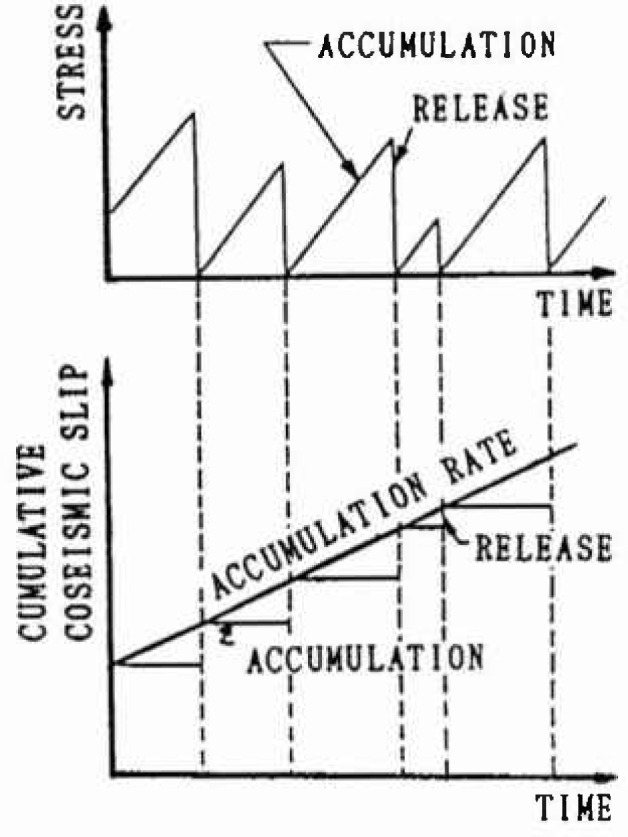
Slip predictable model.

This model, proposed by Shimazaki et al. [4] predicts the size of an earthquake based on the interval times. Figure 2 shows schematically the stress accumulation and release at a fault. For this model, it is assumed that the stress at the fault drops to zero after each earthquake. The time up to the next event is random, and the longer the interval, the greater the event due to the release of the larger stress. Kiremidjian et al. [6] extend the slippredictable model to a site hazard model using the attenuation law. However, the occurrences of successive earthquakes are independent according to the above assumption, and the process of foreshock, mainshock, and aftershock at the same fault cannot be rationally explained.

### 2.3 Semi-Markov Model

This model was proposed by Patwardhan et al. [7]. It is based on the assumption that the size of the earthquake and the interval of time until the next earthquake are influenced by the amount of strain energy released by the previous earthquake. However, a weakness of this model is that subjective assessment is required when classifying the magnitude. That is, the evaluated value is supposed to vary with the classified magnitude because if the magnitude is changed by only 1.0, the released energy varies by about thirty times. Also, the validity of the assumed probability distribution for the time interval is questionable.

As mentioned above, it is necessary to harmonize the stochastic model with the geophysical model of earthquake occurrence for purposes of seismic risk analysis, because the parameters in any model contain some uncertainties. Therefore, we based our research on the theory of plate tectonics [8]. This theory postulates that "the strain energy is accumulated due to the interaction movement of the plates. At the time when the accumulated strain energy reaches a certain extent, an earthquake occurs due to the break of the plates, and the strain energy is released. So, some interval is necessary for the accumulation of strain energy leading to the occurrence of the next event."

In this analysis, it is assumed that the size and the time interval before the occurrence of the next earthquake depend on the strain energy accumulation in the plate at present. A method for forecasting the magnitude of future earthquakes and the distributions of maximum ground acceleration at several main cities in Japan is proposed.

## 3. Seismic Risk Analysis

In this analysis, the focal region which would influence Japan is restricted to latitudes from 25° N to 50° N and longitudes from 125° E to 150° E. This zone is divided into meshes of 0.5° and relative strain energy accumulation in each mesh at present is estimated. Next, in each mesh, the extremal distributions of magnitude of earthquakes which are expected to occur in the next *n* years are estimated. Furthermore, the extremal distributions of maximum ground acceleration at main cities are derived by means of the attenuation law.

Presently, it is difficult to estimate the absolute strain energy accumulation, but seismic risk analysis can be performed by estimating the relative strain energy accumulation, according to the following assumptions.

### 3.1 Earthquake Data

In this analysis, we employ data on earthquakes occurring in or near Japan from 1885 to March 1988 available from the Meteorological Agency [9,10,11,12,13]. However, since the accuracy of methods used in the past to evaluate magnitude is unreliable, the data are corrected by means of the following method [14] proposed by the Ministry of Construction. The method is based on the assumption that "the long-term incline of the curved line of energy accumulation is almost constant and that the incline from 1926 to 1973 shows a value peculiar to Japan." The magnitudes of earthquakes which occurred from 1885 to 1925 are corrected by the following formulas

$$\begin{array}{l} 1885-1895:M=M\prime -0.5\\ 1896-1915:M=M^{\prime }-0.6\\ 1916-1925:M=M^{\prime }-0.5\\ 1926-\;:M=M^{\prime } \end{array}$$ (2)

where

*M* = magnitude before correction
*M′* = magnitude after correction.

We consider that a deep earthquake (focal depth larger than 100 km) does not have much influence on surface ground motion and that plate thickness is approximately 100 km. Therefore, we limited our investigation to earthquakes with a focal depth of 100 km or less occurring after 1926 when focal depth was added to earthquake data.

### 3.2 Fault Model

In general, fault movement is not uniform in either time or space. But fault movement must be simplified for the sake of modeling the earthquake occurrence from a technical viewpoint. So in this analysis, it is assumed that a rectangular fault occurs at the time of earthquake occurrence, that its center agrees with the epicenter, that the ratio of its long side to its short side is 2 : 1, and that a section of the fault is at an angle of 45° with the horizontal plane. Moreover, it is assumed that the long side runs parallel to a longitudinal line if the epicenter is located at latitude from 35° N to 41° N, and parallel to a latitudinal line otherwise [15].

Furthermore, in allowance with the concept of the basic fault model by Kanamori [16], it is supposed that strain energy is released uniformly in proportion to some meshed part of the shadow which the rectangular fault casts on the horizontal plane. In practice, however, the areas releasing the strain energy do not always spread around the epicenter, but stretch in only one direction in many cases. Therefore, with regard to data on such faults included in the earthquake fault parameter handbook in Japan [15] and to enable interpretation of the shapes of the faults, it is assumed that the epicenter agrees with the center of the faults. Concerning the relation between section of a fault and magnitude, the proposed equation by Satou [15] is adopted, and the length of the long side of a fault is determined by the following equation:

logL=0.5M−1.88, (3)

where

*L* = length of the long side of a fault
*M* = magnitude.

A released amount of strain energy is assumed in allowance with the following equation proposed by Gutenberg and Richter.

logE=1.5M+11.8, (4)

where

*E* = released amount of strain energy
*M* = magnitude.

### 3.3 Cluster Division of Each Mesh

It is assumed that the rate of strain energy accumulation is constant regardless of time. In general, there are areas which are similar with respect to the changing conditions of the plates and release conditions of the strain energy. But it is currently difficult to accurately estimate the accumulation and the release of strain energy. In this analysis, therefore, in order to grasp the relative strain energy accumulation in each mesh, each mesh is classified into the following three clusters based on the distribution of the sum of total released energy in each mesh from 1885 to March 1988. This assumption is based on the thinking that it is more rational to apply ergodicity to the meshes in which the released rate of strain energy is almost equal than to all meshes. The cluster division is determined by considering the relationship between the earthquake magnitude and the amount of earthquake data.

$$\begin{array}{c} 1)\;\mathrm{cluster}\;1\;:\;M_{E}⩽7.4\\ 2)\;\mathrm{cluster}\;2\;:\;7.4<M_{E}\\ 3)\;\mathrm{cluster}\;3\;:\;M_{E}>7.7 \end{array}⩽7.7$$ (5)

where *M*_E_ = the magnitude into which the annual average released energy in a mesh from 1885 to March 1988 is converted by Eq. (4).

**Fig. 3:**
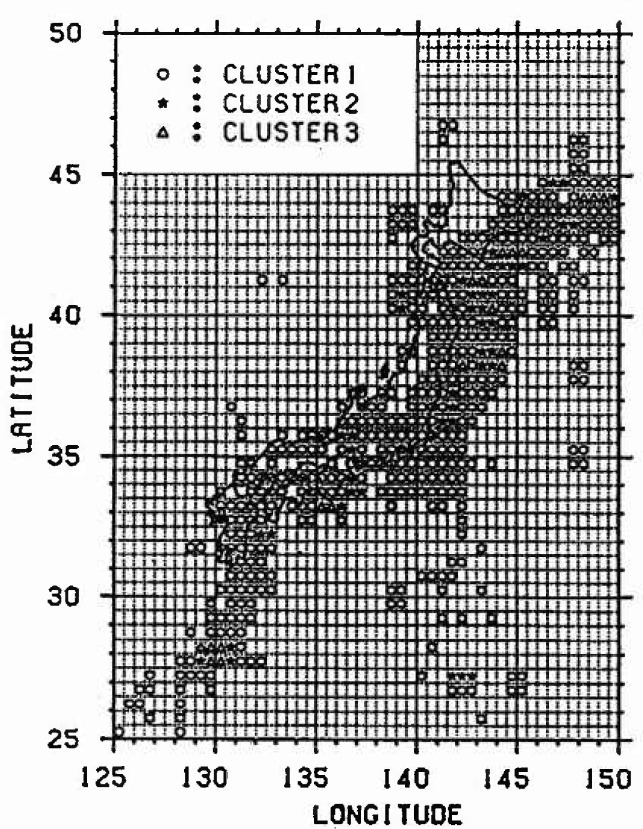
Cluster division of each mesh.

The amount of the annual average released energy for each cluster is averaged, and it is defined as the progress rate of strain energy accumulation. Furthermore, in the case of *M*_E_ <4.5, it is regarded as the strain energy released mainly by the inelastic slip and is not dealt with because the released strain energy is small. The result of classifying each mesh is shown in Fig. 3. The meshes not indicated by marks do not belong to any cluster. From this figure, it is recognized that many earthquakes have occurred along the plates.

### 3.4 Evaluation of the Strain Energy Accumulation in Each Mesh at Present

**Fig. 4:**
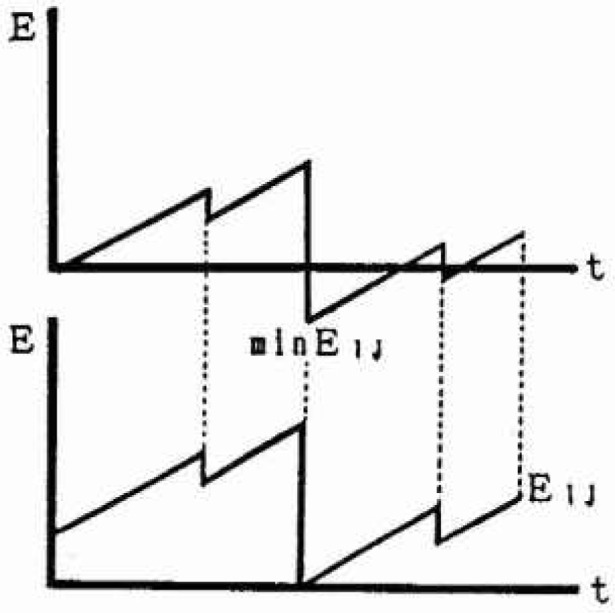
Parallel movement of the strain energy-time curve.

**Fig. 5:**
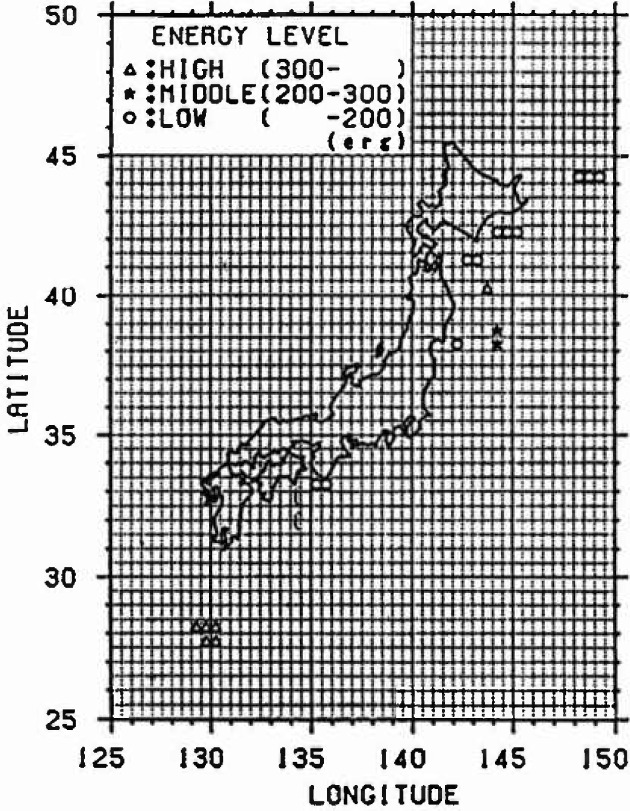
Strain energy accumulation (Cluster 3).

**Fig. 6:**
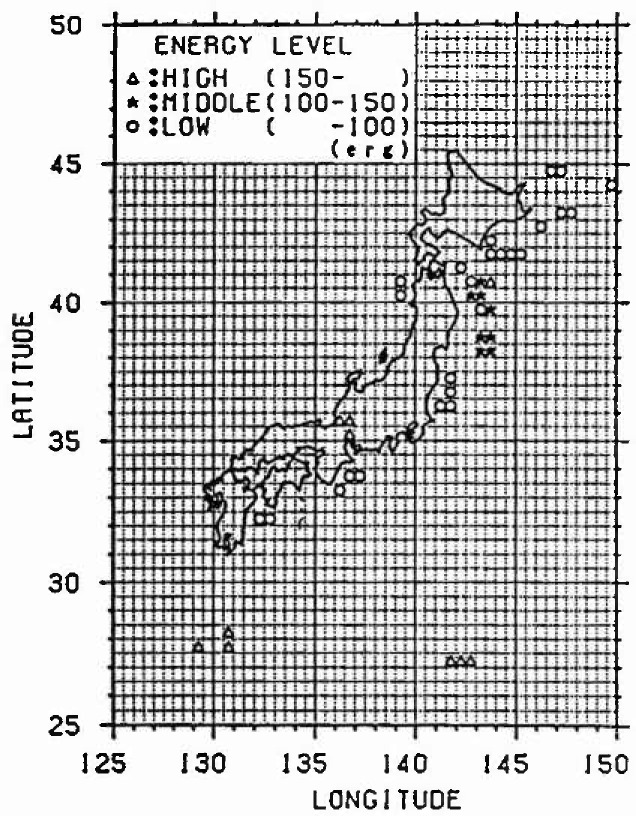
Strain energy accumulalion (Cluster 2).

**Fig. 7:**
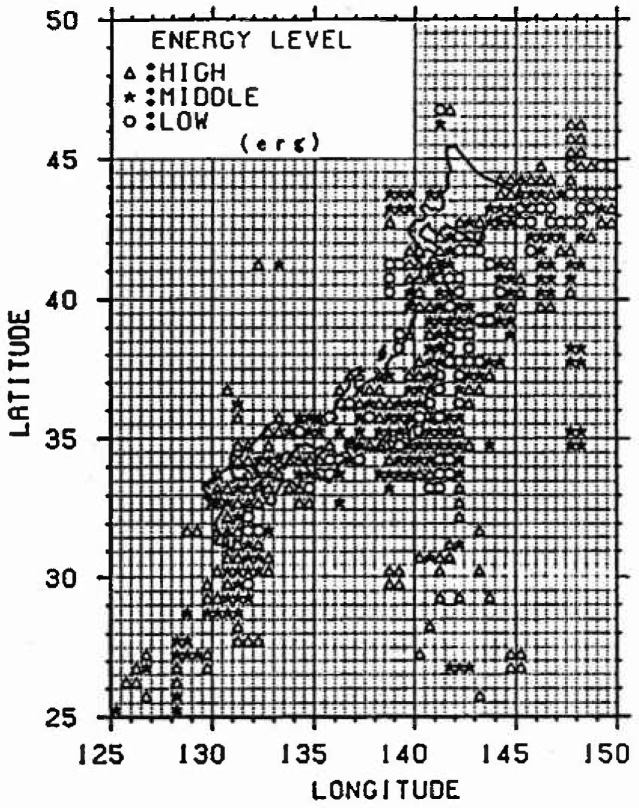
Strain energy accumulation (Cluster 1).

In order to evaluate the relative strain energy accumulation (*E_ij_*) in a mesh (*i j*) with latitude *i*° N and longitude *j*º E as the center, it is necessary to estimate the strain energy accumulation of the plate at the time of occurrence of the oldest earthquake adopted in this analysis. In general, it is supposed that the recurrence period is peculiar to each focal region, but it is difficult to evaluate them strictly at present. Kanamori [8] reported that the average interval time of a great earthquake with a magnitude on the order of 8.0 is about 100 years on the Pacific side and offing. So in this analysis, it is assumed that all strain energy accumulation is released at least once about every 100 years in each mesh. Based on this assumption, the minimum strain energy accumulation (min *E_ij_*) on the strain energy-time curve is regarded as being relative strain energy accumulation 0, and the strain energy-time curve is moved in parallel as shown in Fig. 4. The relative strain energy accumulation (*E_ij_*) in each mesh at present is estimated by the preceding method. The value of the relative strain energy accumulation in each mesh of each cluster is represented in Figs. 5 to 7. In cluster 3, the relative strain energy accumulation is divided into three classes, i.e., high (more than 300 erg), middle (200–300 erg) and low (less than 200 erg). In cluster 2, the accumulation is divided into high (more than 150 erg), middle (100–150 erg) and low (less than 100 erg) (1 erg = 10^7^ joules). As the strain energy accumulations in all meshes of cluster 1 are not high, that cluster is divided into three equal parts.

### 3.5 Evaluation of Extremal Distribution of Magnitude Considering the Strain Energy Accumulation at Present

**Fig. 8:**
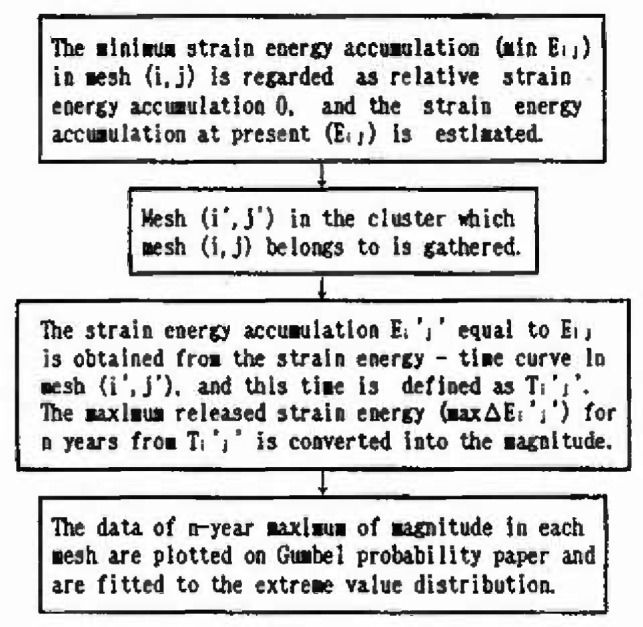
Flow-chart of the analysis.

**Fig. 9:**
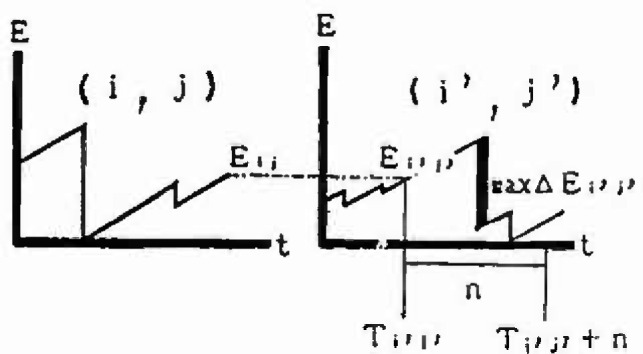
Schematic representation of the analysis.

**Fig. 10:**
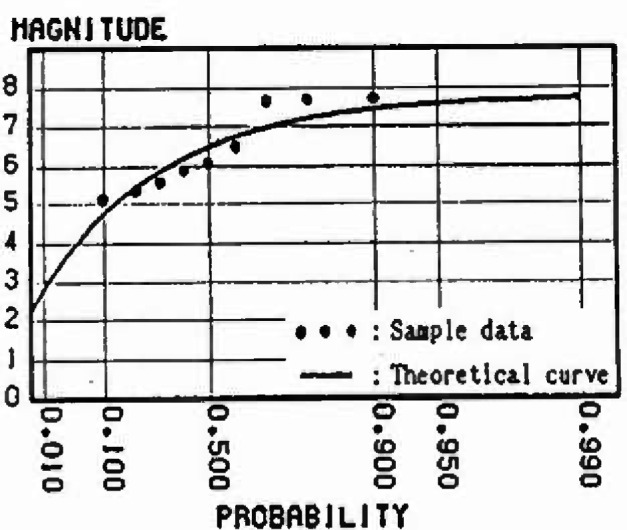
Distribution of 50 year maximum of magnitude (Cluster 3).

**Fig. 11:**
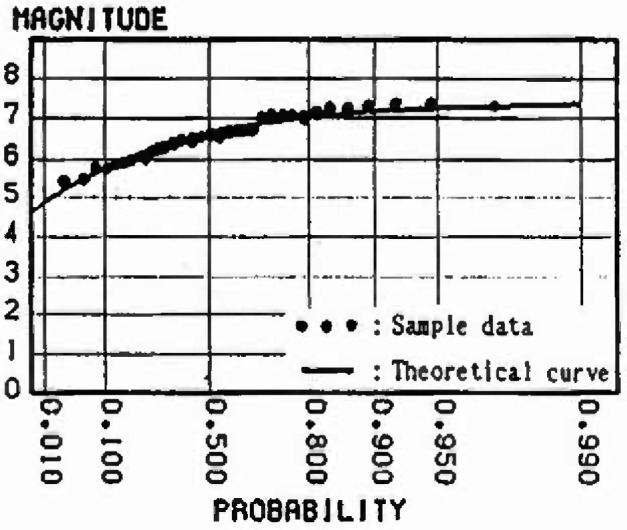
Distribution of 50 year maximum of magnitude (Cluslcr 2, Energy level = Middle).

**Fig. 12:**
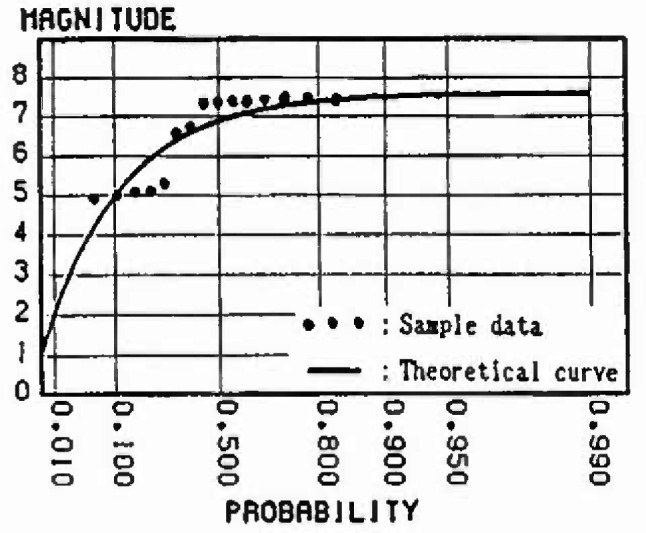
Distribution of 50 year maximum of magnitude (Cluster 2, Energy level = High).

**Fig. 13:**
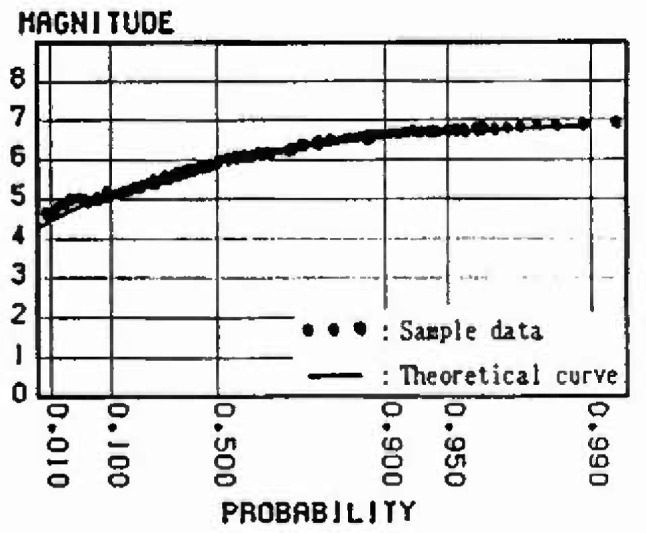
Distribution of 50 year maximum of magnitude (Cluster 1).

In this section, the extremal distributions of magnitude in each mesh are evaluated. The process by which the strain energy is released in allowance with the size of the earthquake, and is again accumulated as time passes, is repeated in each zone. Thus, the strain energy accumulation at present greatly influences the extremal distribution of magnitude of the earthquake expected to occur in the future. If sufficient earthquake data are gathered, it is possible to obtain the extremal distributions of magnitude of each mesh. However, the earthquake data measured by seismographs in Japan are 100 years old at most; the period of observation is not sufficient in light of the recurrence period of great earthquakes. So in this analysis, to evaluate the extremal distributions of magnitude in each mesh, ergodicity is applied to each mesh in the same cluster. Figure 8 shows a flow-chart of the analysis based on this assumption. Figure 9 shows this method schematically. First, the strain energy accumulation of *E_ij_* in a mesh (*i j*) at present is evaluated, and the strain energy accumulation of *E_ij_* equal to *E_ij_* is determined based on strain energy-time curves in other meshes of the same cluster. Next, this time is defined as *T_i j_* and the maximum released strain energy (max∆*E_i j_*) for *n* years from *T_i j_* is converted into the magnitude by Eq. (4). Some samples from each mesh (*i j*) are obtained. To evaluate the form of the distribution, these samples are plotted on the Gumbel probability paper. Assuming that *n* = 50 years, the samples in meshes around Sendai are plotted on the Gumbel probability paper in Figs. 10 to 13. Figure 10 shows the extremal distribution of cluster 3, Figs. 11 and 12 show that of cluster 2 and Fig. 13 shows that of cluster 1. On Gumbel probability paper, the type I extreme value distribution is indicated by a straight line, type II is indicated by a lower convex curve and type III is indicated by an upper convex curve. The upper limit value is decided from maximum sample data rounded off to one decimal and parameters are decided by using the method of least squares.

### 3.6 Evaluation for Extremal Distribution of Maximum Ground Acceleration

Ten cities in Japan where earthquake observatories are situated are chosen as the points for calculating the maximum ground acceleration. The following attenuation law [17] suited for standard clay is proposed by the Public Works Research Institute of the Ministry of Construction and is adopted in this analysis.

$$Acc_{\max}=18.4\times 10^{0.302M}\times \Delta^{-0.8}$$ (6)

where

*Acc*_max_ = maximum ground acceleration
*M* = magnitude
∆ = epicentral distance

The extremal distribution of the maximum ground acceleration is estimated as follows.

- It is assumed that a mesh (*i, j*) is a hypocenter, and the epicentral distance from the center of the mesh to a city is calculated.
- Magnitude *M_ij_* is obtained from the attenuation law for which the epicentral distance and an acceleration *Acc*_max_ are substituted.
  $$M_{ij}=g\left(Acc_{\max},\Delta_{ij}\right)$$ (7)
- The value of the distribution function *F_aij_*(*a*) at a maximum ground acceleration *Acc*_max_ is evaluated by using the shape parameter, the modal value and the characteristic largest value of the extremal distribution of the magnitude in the mesh ((*i,j*), and *M_ij_* is obtained as in b).
- The previous operation is done for each mesh for maximum ground acceleration. Then using the following equation, the distribution function at city *F_a_*(*a*) is obtained.
  $$F_{a}\left(a\right)=\prod_{i=1}^{n}\prod_{j=1}^{m}F_{aij}\left(a\right)$$ (8)
- *F_a_* (*a*) is obtained by the preceding operation from b) to d) for some (*Acc*_max_) accelerations, and the relation between *F_a_*(*a*) and *a* is plotted on Gumbel probability paper.

**Fig. 14:**
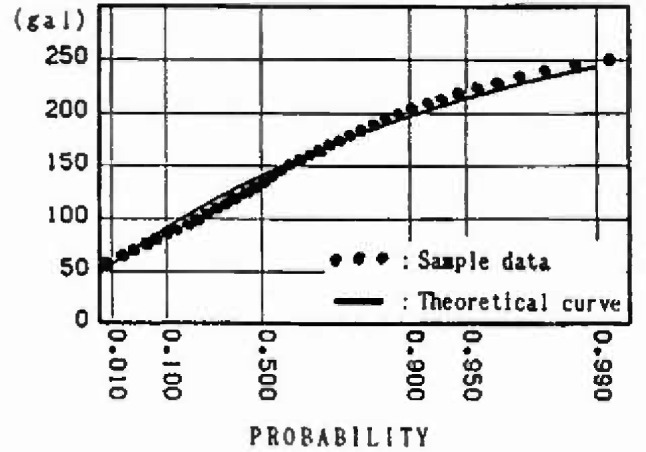
Distribution of 50 year maximum of maximum ground acceleration at Sendai.

**Fig. 15:**
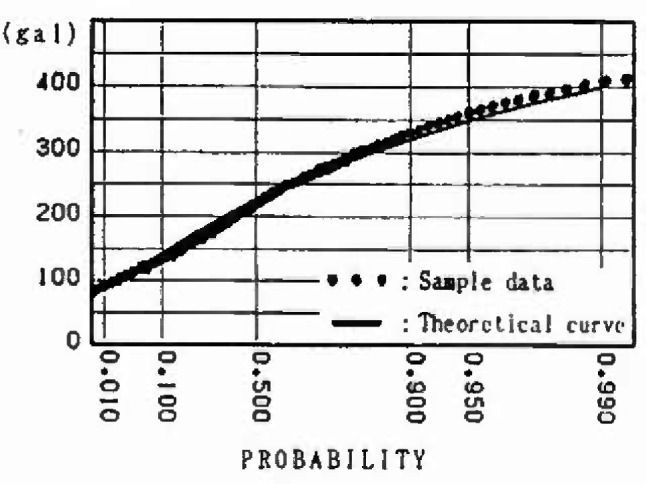
Distribution of 50 year maximum of maximum ground acceleration at Tokyo.

Thus, the extremal distributions of the maximum ground acceleration at main cities are obtained. Figures 14 and 15 show the distribution for 50 year maximum of maximum ground acceleration at Sendai and Tokyo, respectively. Moreover, in order to examine the nonstationarity, the expected values and the coefficients of variation of the distributions for 50, 40, 30, 20, and 10 year maximums of maximum ground acceleration in 1988 are shown in Table 1. Furthermore, the expected values and the coefficients of variation of the distributions for the 50 year maximum of maximum ground acceleration at the different starting points (1968 and 1988) are shown in Table 2.

## 4. Results and Considerations

### 4.1 Relative Strain Energy Accumulation

Figures 5 to 7 show the relative strain energy accumulation in each cluster at present. It can be recognized that most meshes in each cluster are distributed near the boundary of the plate of the Pacific side in Kanto, Tohoku, and Hokkaido, and many earthquakes occur in those places. Moreover, it can be assumed that a large earthquake is likely to occur in places in which the strain energy Accumulation is high such as in cluster 3 at present.

### 4.2 Extremal Distribution of the Magnitude in Each Mesh

The distributions for the 50 year maximum of magnitude in the meshes near Sendai are shown in Figs. 10 to 13. Judging from theoretical curve of the type III extreme value distribution, the data accounting for the relative strain energy accumulation at present obviously fit this distribution. The probability of the occurrence of a large earthquake is greater as the strain energy accumulation at present increases. For example, comparing Fig. 11 with Fig. 12, which show the distribution for the 50 year maximum of magnitude in the meshes at cluster 2, the magnitude at a probability exceeding 0.2 is less than 7.5 in Fig. 12 and 7.0 in Fig. 11, respectively, because the strain energy accumulation at present in Fig. 12 is higher than that in Fig. 11.

Therefore, it is thought that the form of the distribution of the 50 year maximum of magnitude remains unchanged, but that the magnitude at the probability of occurrence varies depending on the strain energy accumulation at present.

### 4.3 Extremal Distribution of Maximum Ground Acceleration at Main Cities

**Fig. 16:**
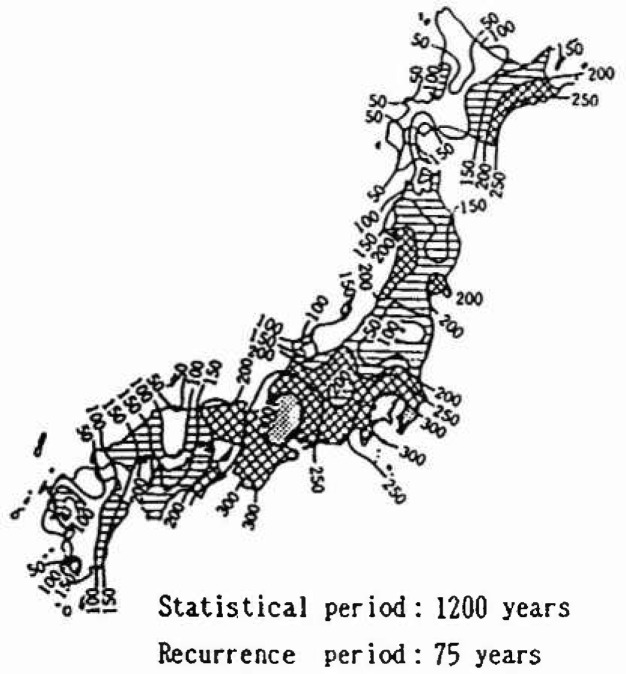
Seismic risk map by Gotou and Karneda.

The distributions for the 50 year maximum of maximum ground acceleration at main cities are shown in Figs. 14 and 15. Those distributions fit the type III extreme value distribution as do the distributions for the 50 year maximum of magnitude. The expected values and the coefficients of variation of the distributions for 50, 40, 30, 20, and 10 year maximums of maximum ground acceleration in 1988 are shown in Table 1. Those values reflect the strain energy accumulation at present in the mesh in which the cities are located. Comparing the expected values for *n* = 50 years in Table 1 with the seismic risk map of maximum ground acceleration by Gotou and Kameda [18] in Fig. 16, the expected values yielded by this analysis for Kyoto and Osaka are extremely low. Extensive earthquake data were available for the Kyoto area in which population and culture have been concentrated; the analysis by Gotou and Kameda used historical earthquake data based on estimations from the ancient records.

### 4.4 Examination of Nonstationarity in Maximum Ground Acceleration

According to Table 1, the expected values of extremal distribution of maximum ground acceleration at Sapporo and Niigata are almost constant from *n* = 10 years to *n* = 50 years because the seismicities of these cities are not active. However, in other cities, there are large differences in the expected values between *n* = 10 years and *n* = 50 years; in particular difference in Tokyo is 40 gal (1 gal = 1 cm s^−2^). Moreover, the expected values and the coefficients of variation of the distributions for the 50 year maximum of maximum ground acceleration at the different starting points (1968 and 1988) are shown in Table 2. According to Table 2, the difference of the expected values in Niigata and Nagoya are small, but about 10 gal in Tokyo and Sendai, and 40 gal in Osaka. So, it is recognized that the expected value of maximum ground acceleration varied due to the strain energy accumulation at that time. Therefore, it is necessary to consider the nonstationarity in earthquake occurrence when determining the earthquake load in design.

## 5. Conclusions

This analysis employs seismic risk analysis in which the focal regions which would have an influence on Japan were restricted. This zone was divided by meshes with 0.5° angles, and relative strain energy accumulation in each mesh was estimated by taking account of the nonstationarity in earthquake occurrence. The distributions for the 50 year maximum of magnitude in each mesh were evaluated. Furthermore, the extremal distributions of maximum ground acceleration at the main cities were derived by means of the attenuation law. From this analysis, the following conclusions can be stated:

1. A procedure of seismic risk analysis taking account of the relative strain energy accumulation was proposed.
2. The distributions for the 50 year maximum of magnitude in each mesh fitted the type III extreme value distribution very well.
3. As the strain energy accumulation at present increases, the value of magnitude at a probability of occurrence becomes greater.
4. The distributions for the 50 year maximum of maximum ground acceleration at main cities also fitted the type III extreme value distribution.
5. The expected value of maximum ground acceleration at a city reflected the strain energy accumulation at present in the mesh in which the city is located.
6. this analysis is capable of forecasting the earthquake load suited to the service life of a structure, That is, it is possible to determine a more rational earthquake load in design by estimating the strain energy accumulation at the time when the structure will be constructed.
7. This analysis is capable of evaluating the extremal distributions for maximum ground acceleration and those expected values in all parts of Japan, and it seems that these statistics are useful for the criterion of aseismic design.

As mentioned above, this seismic risk analysis is capable of taking account of the nonstationarity in earthquake occurrence by estimating the strain energy accumulation in each mesh at present. So, with this analysis, it is possible to forecast earthquakes by adopting new earthquake data and to estimate the earthquake load suited to the service life of a structure. However, the data on large earthquakes with recurrence periods of 200 to 300 years are probably insufficient because the earthquake data of the past 100 years in Japan as measured by seismograph are used in this analysis. In this analysis, the seismicity gaps are not treated and the attenuation law is used to cope with standard clay. For obtaining more accurate findings, it is necessary that the attenuation law be suited to each place and condition of clay.

## Figures and Tables

**Table 1:** The expected values and the coefficients of variation of the extremal distributions of maximum ground acceleration at main cities (1 gal = 1 cm s^−2^)

Name of City	Statistics	10 year	20 year	30 year	40 year	50 year
Sapporo	Mean value (gal)	41	42	45	46	46
	COV^a^(%)	16.2	15.9	15.6	15.4	14.6
Sendai	Mean value (gal)	127	129	139	140	143
	COV (*%*)	28.7	28.5	28.5	28.3	28.8
Tokyo	Mean value (gal)	189	199	211	224	229
	COV (%)	29.1	29.9	30.0	29.9	30.0
Niigata	Mean value (gal)	52	53	57	58	59
	COV (%)	21.9	22.3	20.7	19.9	19.4
Nagoya	Mean value (gal)	260	260	262	263	263
	COV (%)	5.4	5.4	3.6	3.9	3.9
Kyoto	Mean value (gal)	87	90	91	93	94
	COV (%)	10.6	11.9	12.2	13.1	13.5
Osaka	Mean value (gal)	85	94	94	99	100
	COV (%)	28.9	24.7	27.7	28.3	28.2
Hiroshima	Mean value (gal)	83	96	99	104	108
	COV (%)	23.0	27.2	25.3	26.2	25.2
Takamatsu	Mean value (gal)	107	110	123	124	129
	COV (%)	32.4	30.1	28.4	28.2	27.0
Fukuoka	Mean value (gal)	93	104	117	133	137
	COV (%)	23.4	23.7	22.1	22.4	22.0

a COV: Coefficient of Variation.

**Table 2:** The expected values and the coefficients of variation of the extremal distributions of maximum ground acceleration in 1968 and 1988 (1 gal = 1 cm s^−2^)

Name of City	Statistics	1968	1988
Sapporo	Mean value (gal)	49	46
	COV^a^(%)	12.9	14.6
Sendai	Mean value (gal)	151	143
	COV (%)	29.9	28.8
Tokyo	Mean value (gal)	239	229
	COV (%)	30.1	30.0
Niigata	Mean value (gal)	58	59
	COV (%)	18.0	19.4
Nagoya	Mean value (gal)	261	263
	COV (%)	3.8	3.9
Kyoto	Mean value (gal)	96	94
	COV (%)	12.4	13.5
Osaka	Mean value (gal)	139	100
	COV (%)	16.0	28.2
Hiroshima	Mean value (gal)	117	108
	COV (%)	20.3	25.2
Takamatsu	Mean value (gal)	136	129
	COV (%)	21.9	27.0
Fukuoka	Mean value (gal)	144	137
	COV (%)	17.6	22.0

a COV: Coefficient of Variation.
